# Prevalence of Cancer in Patients with Venous Thromboembolism: A Retrospective Nationwide Case-Control Study in Sweden

**DOI:** 10.1177/10760296231158368

**Published:** 2023-02-27

**Authors:** Katarina Glise Sandblad, Per-Olof Hansson, Jacob Philipson, Ahmad Mahmoud, Per Karlsson, Annika Rosengren, Jan Sörbo

**Affiliations:** 1 Department of Molecular and Clinical Medicine, Institute of Medicine, Sahlgrenska Academy, University of Gothenburg, Sahlgrenska University Hospital/Östra, Gothenburg, Sweden; 2 Department of Medicine, Geriatrics and Emergency Medicine, Region Västra Götaland, Sahlgrenska University Hospital/Östra, Gothenburg, Sweden; 3 Department of Oncology, Institute of Clinical Sciences, Sahlgrenska Academy, Sahlgrenska University hospital/ Sahlgrenska, Gothenburg University, Gothenburg, Sweden; 4 Department of Clinical Physiology, Region Västra Götaland, Sahlgrenska University Hospital/Östra, Gothenburg, Sweden

**Keywords:** (MESH): incidence, neoplasms, registry, sex difference, venous thromboembolism

## Abstract

Cancer is a risk factor for venous thromboembolism (VTE). We aimed to define sex-specific risk of preceding cancer in patients with a first-time VTE by conducting a nationwide Swedish registry-based study including 298 172 patients with VTE and 1 185 079 matched controls. This included 44 685 patients with a diagnosis of cancer at/or within 1 year before a VTE diagnosis. Female patients with VTE had a higher multivariable adjusted odds ratios of preceding cancer than male patients with VTE (5.5 [99% confidence interval 5.4-5.7] vs 3.9 [3.8-4.0]). The highest risk of cancer in patients with VTE were found for pancreatic cancer (women: 19.6 [15.8-24.4]; men: 17.2 [13.7-21.6]) and brain cancer (women: 17.4 [12.9-23.4]; men: 17.5 [13.8-22.2]). Weak associations were seen between VTE and bladder/urothelial cancer (women: 1.31 [1.12-1.53]; men: 1.34 [1.23-1.47]), prostate cancer (men: 2.17 [2.07-2.27]), malignant melanoma (women: 2.51 [2.07-3.05]; men: 2.67 [2.23-3.18]), and kidney cancer (women: 3.20 [2.49-4.11]; men: 3.33 [2.79-4.07]). In conclusion, associations with VTE were weak for bladder/urothelial cancer and kidney cancer, and strong for pancreatic, brain, and biliary cancers.

## Introduction

Cancer is a strong risk factor for venous thromboembolism (VTE).^1^ Patients with cancer who suffer VTE have shorter overall survival^2^ and increased healthcare costs,^3^ and often display physical and emotional distress.^4^ VTE in patients with cancer can also lead to delayed antitumoral treatment^5^ and decreased quality of life.^6^

In a previous large Swedish register-based study on comorbidities and temporary provoking factors for VTE, we found that a diagnosis of cancer had the highest population-attributable risks for both pulmonary embolism (PE) and deep vein thrombosis (DVT).^7^ However, the association between VTE and different cancer types warrants further investigation. It is well known that patients with different cancer types have varying risks of VTE, but previous studies report different magnitudes of VTE risk.^1,8–11^

In the present nationwide study, we aimed to determine the incidence of VTE in association with a recent diagnosis of cancer and provide information on the sex and age distributions for both all cancers and separate cancer types in patients with VTE. We also aimed to estimate sex-specific odds ratios (ORs) for various cancers in patients with VTE.

## Methods

### Study Population

**Figure 1.**
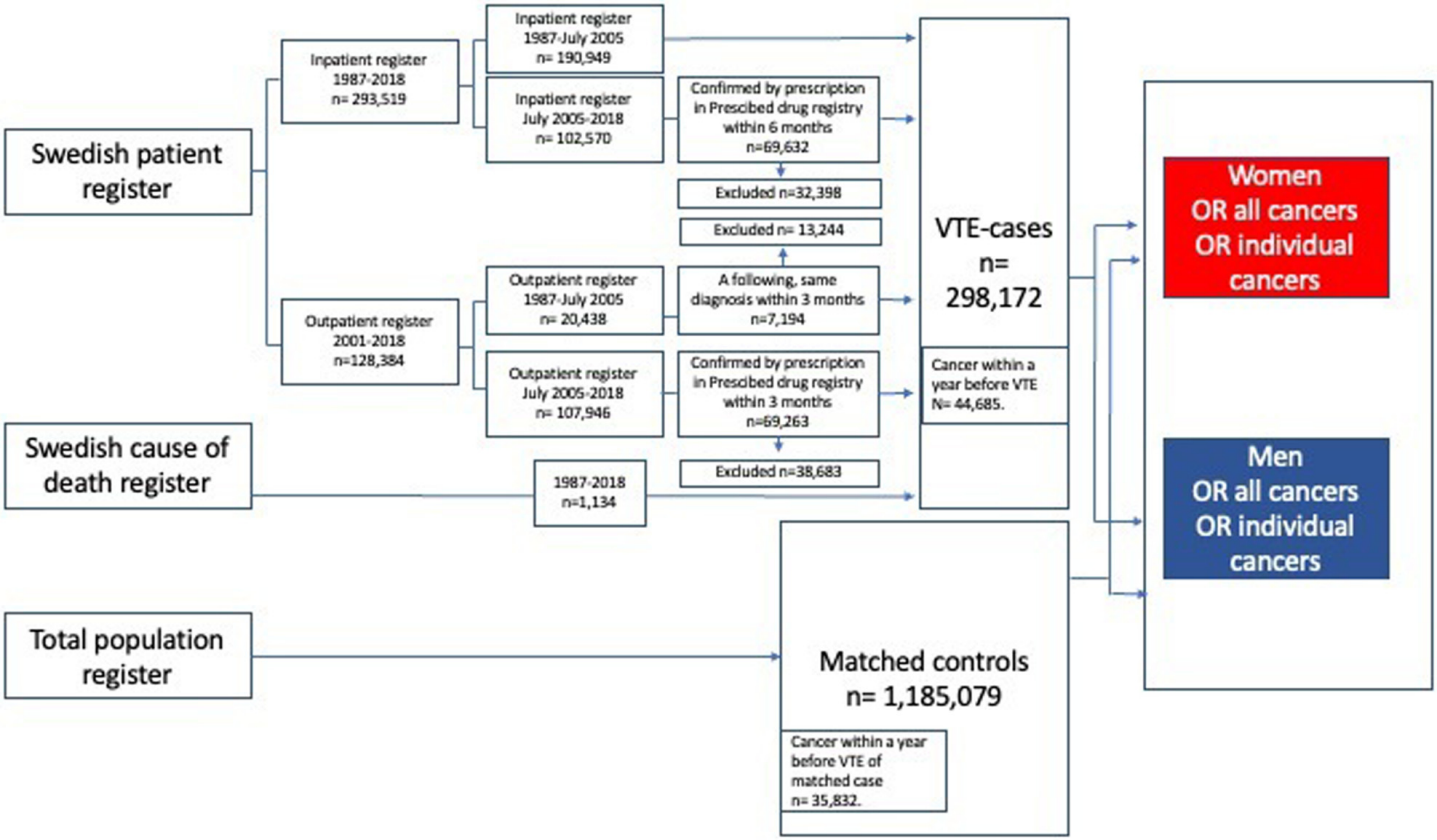
Included registries and their contributions to the total number of VTE cases and matched controls. Abbreviation: VTE, venous thromboembolism.

This case-control study was based on 4 Swedish nationwide health and administrative registries: Swedish Patient Register, Swedish Cause of Death Register, Prescribed Drug Register, and Total Population Register (Figure 1). Detailed information on the registries is provided in Supplemental Table 1. Entries in the registries are linked by the Swedish Personal Identifier Number (PIN), a unique number that identifies every Swedish citizen. Sweden has a publicly financed healthcare system that provides low-cost outpatient and inpatient hospitalcare for all citizens. Patients with PE and DVT in Sweden are almost exclusively diagnosed and treated in hospital-based clinics, rather than in primary care. Hence, the Swedish Patient Register has virtually complete coverage of VTE cases in individuals with a Swedish PIN.

We identified all cases with first-time VTE between 1987 and 2018 and selected up to 4 controls from the Total Population Register, matched by sex, year of birth, and county of residence. The controls were free of registered VTE on the date of inclusion, which was the date of VTE for the matching case. However, a control could subsequently be registered with VTE and then be registered as a case. Both cases and controls could have a diagnosis of cancer before inclusion. To identify first-time VTE events as much as possible, we excluded cases and controls with a registered PE or DVT diagnosis between January 1, 1980 and December 31, 1986. After linkage, all PINs were replaced by codes.

VTE was defined using International Classification of Diseases codes as follows: Revision 8 (ICD8): PE (450), DVT (451); Revision 9 (ICD9): PE (415B, 416W), DVT (451 except 451A); Revision 10 (ICD10): PE (I26), DVT (I80 except I80.0). Both primary and secondary diagnoses for cause of hospitalization or death were accepted. The case selection is shown in Figure 1. VTE cases registered before July 1, 2005 were defined as follows: (1) single first inpatient VTE diagnosis; (2) first outpatient VTE diagnosis and subsequent same diagnosis within 3 months; or (3) single first VTE diagnosis in the Cause of Death Register. After July 1, 2005, VTE cases were defined as follows: (1) single first inpatient VTE diagnosis *and* retrieval of anticoagulant drug within 6 months; (2) single first outpatient VTE diagnosis *and* retrieval of anticoagulant drug within 3 months; or (3) single first VTE diagnosis in the Cause of Death Register. If more than one diagnosis was registered for the same patient on one date, outpatient diagnoses were counted before inpatient diagnoses, and cause of death diagnoses were counted last.

VTE events with inpatient or outpatient recording of a diagnostic code of cancer within 1 year before or on the same date as the VTE diagnosis were included in the analysis. For the controls, recording of a diagnostic code of cancer within 1 year before or on the same date as the VTE diagnosis in the matched case was used. The included cancers and their diagnostic codes are listed in Supplemental Table 2. Cancer in situ was not included in the analysis.

Comorbidities registered within 1 year before or on the same date as the VTE and temporary provoking factors registered within 3 months before or on the same date as the VTE were used for calculation of multivariable adjusted ORs (aORs) of various cancers in patients with VTE. Comorbidities included heart failure, ischemic heart disease, atrial fibrillation/flutter, ischemic stroke, hemorrhagic stroke, chronic obstructive pulmonary disease, and inflammatory bowel disease. Temporary provoking factors included major surgery, trauma, and lower extremity fracture. The ICD codes for various for comorbidities and temporary provoking factors in Supplemental Table 3.

### Statistical Analysis

Categorical variables are presented as number and percentage. Continuous variables are presented as mean ± standard deviation and median with interquartile range.

Sex-stratified and age-stratified incidence rates were calculated by dividing VTE cases with cancer with the total amount of inhabitants in the Swedish population of the same age group in each calendar year, using population data from the Statistical Database compiled by Statistics Sweden. The total incidence rate was calculated as the total number of VTE cases with cancer divided by the total person-years at risk (total number of inhabitants in Sweden each calendar year). The percentage of VTE cases with each cancer type was determined by dividing the number of VTE cases in each cancer type by the total number of VTE cases with cancer.

The aORs and 99% confidence intervals (CIs) of VTE for various cancer types were calculated for men and women using conditional logistic regression after case-control matching with adjustment for comorbidities and temporary provoking factors.

All statistical analyses were performed using SAS version 9 for Windows (SAS Institute Inc., Cary, NC, USA).

## Results

### Study Population

A total of 298 172 patients with VTE were registered. Of these, 44 685 (15.0%) had a cancer diagnosis registered within 1 year before or on the same date as the VTE. Among these, 8518 (19.1%) had their first recorded VTE as outpatients, 35 747 (80.0%) as inpatients, and 420 (0.9%) in the Cause of Death Register. Among VTE cases, 22 000 (49.2%) had DVT and 22 685 (50.8%) had PE. When a DVT and a PE were registered on the same date, it was regarded as a PE. The median age at VTE diagnosis was 71 years for the total cohort, 74 years for women, and 69 years for men. The VTE cases were matched with 1,185,079 controls, of whom 35 832 (3.0%) had a cancer diagnosis within 1 year before or on the same date as the VTE in the matched case. The frequencies and proportions of cancer among the VTE cases and controls are shown in Table 1.

**Table 1.** Frequencies and Proportions of Cancer Cases Among the VTE Cases and Controls in the Total Cohort and Stratified by Sex.

	Study cohort					
	All		Women		Men	
	cases	Controls	Cases	Controls	Cases	Controls
	(n = 298 172)	(n = 1 185 079)	(n = 157 244)	(n = 625 117)	(n = 140 928)	(n = 559 962)
Age (y), mean (SD), and median (IQR)	68.1 (16.2) 71 (59-80)	68 (16) 71 (59-80)	69.6 (16.9) 74 (61-82)	70 (17) 74 (61-82)	66.4 (15.2) 69 (57-78)	66 (15) 69 (57-78)
	n (%)	n (%)	n (%)	n (%)	n (%)	n (%)
All cancers except nonmelanoma skin cancer	44 685 (15.0)	35 832 (3.0)	22 347 (14.2)	14 513 (2.3)	22 338 (15.9)	21 319 (3.8)
Brain	1867 (0.6)	253 (0.0)	738 (0.5)	102 (0.0)	1129 (0.8)	151 (0.0)
Pancreatic	1959 (0.7)	375 (0.0)	1082 (0.7)	197 (0.0)	877 (0.6)	178 (0.0)
Liver	624 (0.2)	216 (0.0)	310 (0.2)	84 (0.0)	314 (0.2)	132 (0.0)
Biliary	730 (0.2)	172 (0.0)	498 (0.3)	101 (0.0)	232 (0.2)	71 (0.0)
Lung	4651 (1.6)	1515 (0.1)	2298 (1.5)	701 (0.1)	2353 (1.7)	814 (0.1)
Ovarian	1764 (1.1)	631 (0.1)	1764 (1.1)	631 (0.1)	0 (0.0)	0 (0.0)
Breast	5265 (1.8)	4271 (0.4)	5208 (3.3)	4244 (0.7)	57 (0.0)	27 (0.0)
Esophageal, stomach	1510 (0.5)	613 (0.1)	576 (0.4)	220 (0.0)	934 (0.7)	393 (0.1)
Multiple myeloma	1199 (0.4)	828 (0.1)	525 (0.3)	424 (0.1)	674 (0.5)	404 (0.1)
Testicular	214 (0.2)	107 (0.0)	0 (0.0)	0 (0.0)	214 (0.2)	107 (0.0)
Lymphoma	2590 (0.9)	2034 (0.2)	1163 (0.7)	938 (0.2)	1427 (1.0)	1096 (0.2)
Cervix	601 (0.4)	274 (0.0)	601 (0.4)	274 (0.0)	0 (0.0)	0 (0.0)
Colon	3948 (1.3)	2110 (0.2)	2064 (1.3)	1103 (0.2)	1884 (1.3)	1007 (0.2)
Rectal, anal	2234 (0.7)	1363 (0.1)	984 (0.6)	619 (0.1)	1250 (0.9)	744 (0.1)
Leukemia	1728 (0.6)	1576 (0.1)	796 (0.5)	690 (0.1)	932 (0.7)	886 (0.2)
Small intestinal	282 (0.1)	204 (0.0)	131 (0.1)	94 (0.0)	151 (0.1)	110 (0.0)
Uterine	1508 (1.0)	1188 (0.2)	1508 (1.0)	1188 (0.2)	0 (0.0)	0 (0.0)
Kidney	813 (0.3)	653 (0.1)	326 (0.2)	259 (0.0)	487 (0.3)	394 (0.1)
Malignant melanoma	850 (0.3)	1178 (0.1)	390 (0.2)	558 (0.1)	460 (0.3)	620 (0.1)
Prostate	7169 (5.1)	10 857 (1.9)	0 (0.0)	0 (0.0)	7169 (5.1)	10 857 (1.9)
Bladder, urothelial	2572 (0.9)	3916 (0.3)	643 (0.4)	951 (0.2)	1929 (1.4)	2965 (0.5)

For comparisons between groups, the chi-square test was used for nonordered categorical variables.

Abbreviations: IQR, interquartile range; VTE, venous thromboembolism.

### Incidence Rate of VTE With Preceding Cancer by Age in the Swedish Population

**Figure 2.**
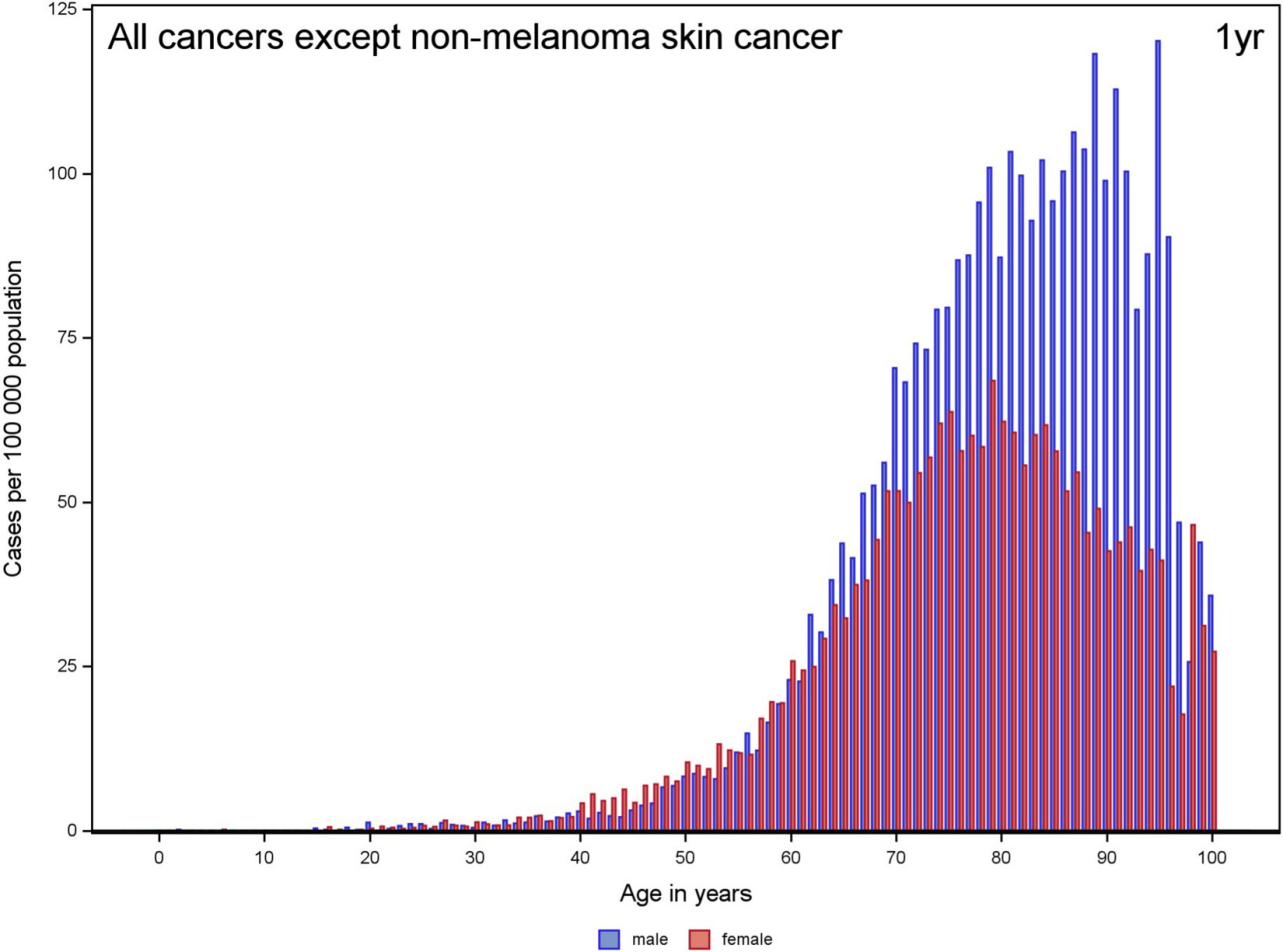
Incidence of patients with VTE with a diagnosis of cancer at or within 1 year before per 100 000 inhabitants in the Swedish population, presented by age and sex. Abbreviation: VTE, venous thromboembolism.

**Figure 3.**
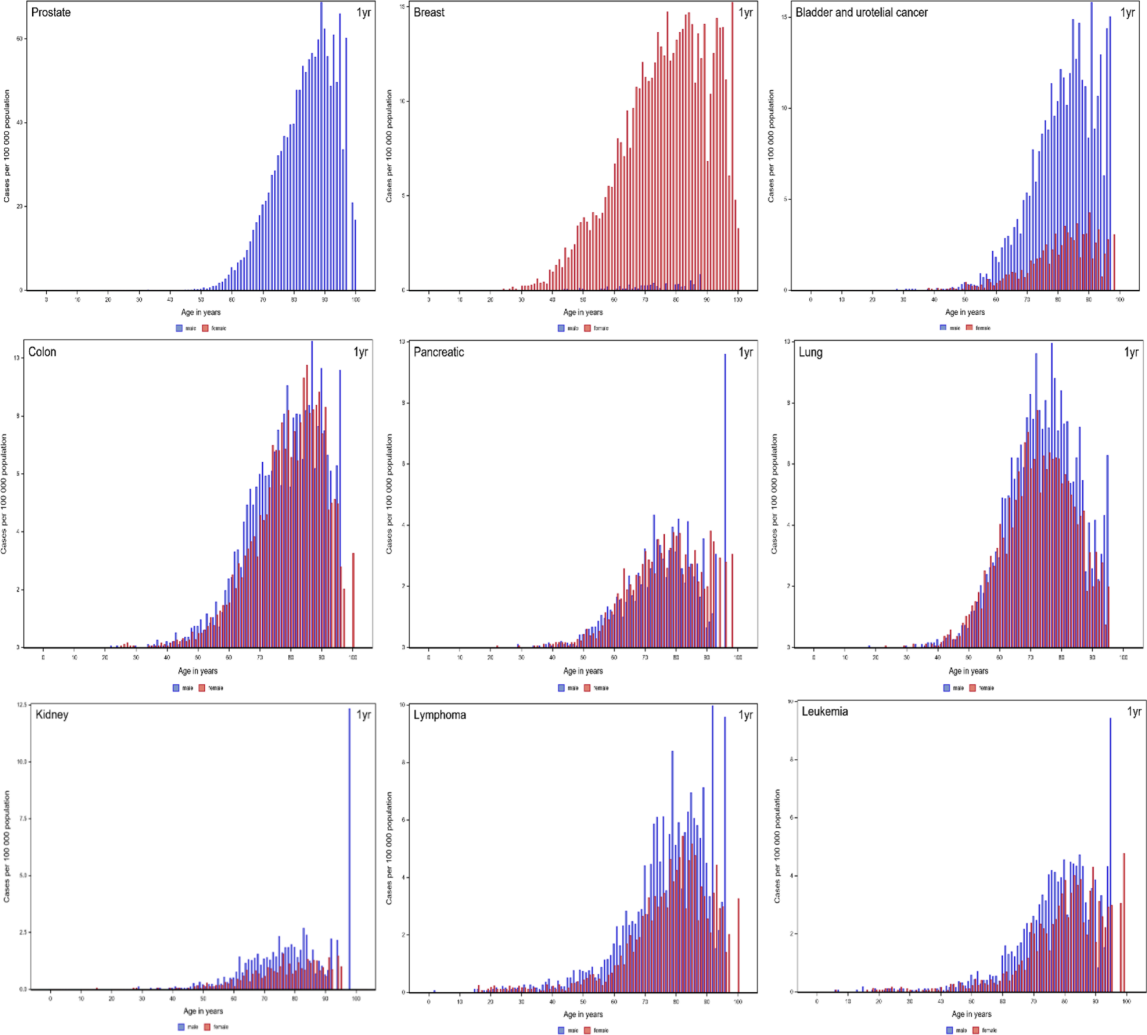

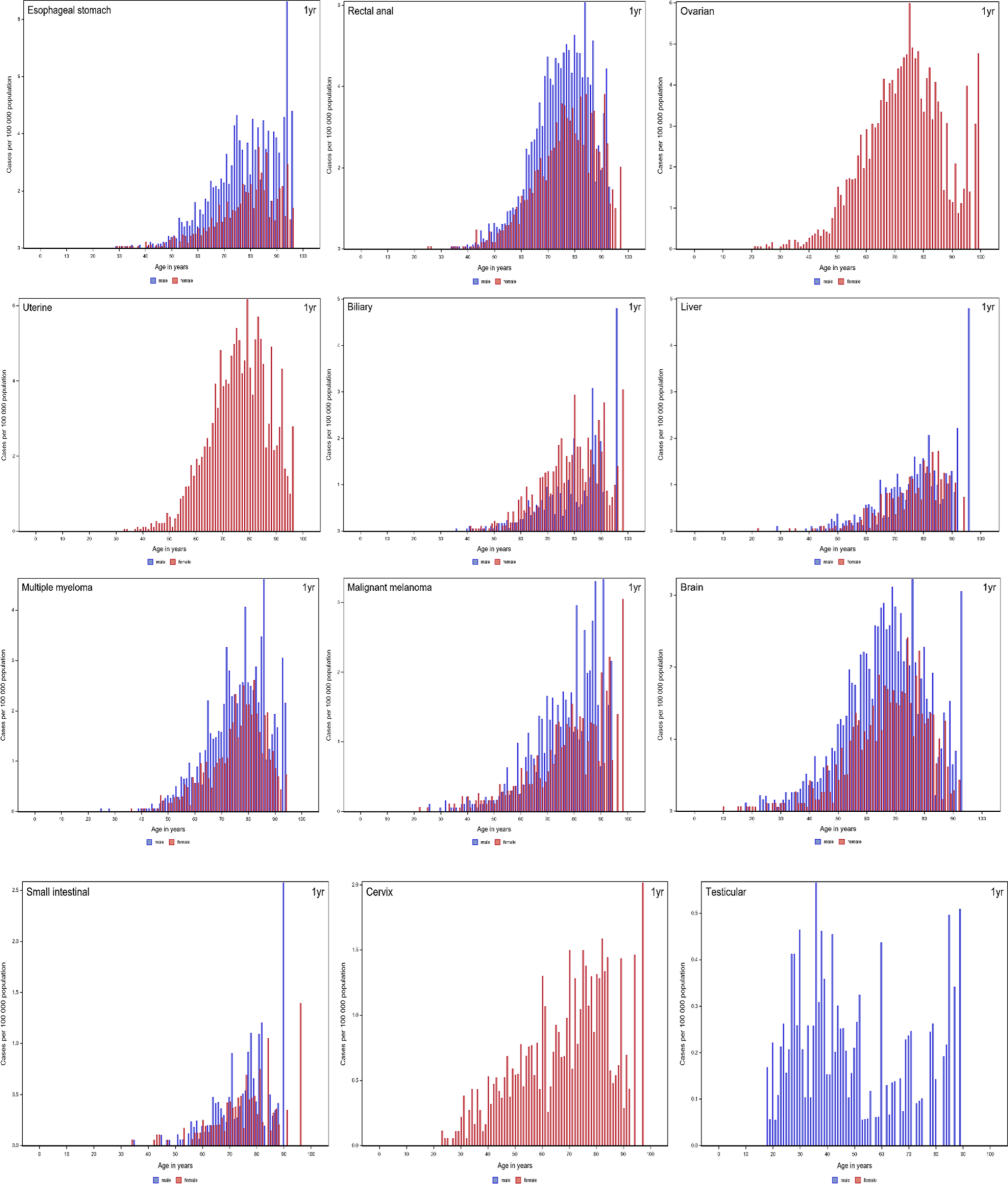
Age-specific and sex-specific incidence rates of VTE with a cancer diagnosis (at or within 1 year before the date of VTE diagnosis) on a population level for various types of cancer. Due to the sharply differing incidence rates, the scales of the graphs are different. Abbreviation: VTE, venous thromboembolism.

The annual incidence of VTE with cancer was 15.8 per 100 000 for the entire population, 15.9 per 100 000 for men, and 15.7 per 100 000 for women, increasing steeply after 60 years of age, particularly in men. Women had a slightly higher incidence between the third and sixth decades of life (Figure 2, Supplemental Table 4). Higher incidence of VTE with cancer in men compared with women was particularly seen for bladder/urothelial, esophageal/stomach, rectal/anal, and brain cancers (see Figure 3).

Among all VTE cases with cancer, the most prevalent cancer types were prostate cancer (16.0%), breast cancer (11.8%), and lung cancer (10.4%) (Table 2). Among female VTE cases, breast cancer (23.3%), lung cancer (10.3%), and colon cancer (9.2%) were most common. Among male VTE cases with cancer, prostate cancer (32.1%), lung cancer (10.5%), and bladder/urothelial cancer (8.6%) were most common.

**Table 2.** Number and Percentage of Specific Cancers in VTE Cases, Stratified by Sex.

	All		Women		Men	
	VTE, n	VTE, %	VTE, n	VTE, %	VTE, n	VTE, %
All cancers except nonmelanoma skin cancer	44 685		22 347		22 338	
Brain	1867	4.20	738	3.30	1129	5.05
Pancreatic	1959	4.38	1082	4.84	877	3.93
Liver	624	1.40	310	1.39	314	1.41
Biliary	730	1.63	498	2.23	232	1.04
Lung	4651	10.41	2298	10.28	2353	10.53
Ovarian	1764	3.95	1764	7.89	0	0.00
Breast	5265	11.78	5208	23.31	57	0.26
Esophageal, stomach	1510	3.38	576	2.58	934	4.18
Multiple myeloma	1199	2.68	525	2.35	674	3.02
Testicular	214	0.48	0	0.00	214	0.96
Lymphoma	2590	5.80	1163	5.20	1427	6.39
Cervix	601	1.34	601	2.69	0	0.00
Colon	3948	8.84	2064	9.24	1884	8.43
Rectal, anal	2234	5.00	984	4.40	1250	5.60
Leukemia	1728	3.87	796	3.56	932	4.17
Small intestinal	282	0.63	131	0.59	151	0.68
Uterine	1508	3.37	1508	6.75	0	0.00
Kidney	813	1.82	326	1.46	487	2.18
Malignant melanoma	850	1.90	390	1.75	460	2.06
Prostate	7169	16.04	0	0.00	7169	32.09
Bladder, urothelial	2572	5.76	643	2.88	1929	8.64

“All cancers” includes some cancer types that are not accounted for in the separate cancer groups, see Supplemental Table 2.

Individuals could have more than one registered cancer diagnosis.

Abbreviation: VTE, venous thromboembolism.

### Unadjusted Odds Ratios of Different Cancer Types in Patients With VTE Compared to Matched Controls Without VTE

In females, the strongest association with VTE was seen for brain (OR: 28.9), pancreatic (OR: 22.5), biliary (OR: 19.8), and liver cancers (OR: 16.5). Among males brain (OR: 31.3), pancreatic (OR: 20.9), biliary (OR: 14.0), and lung cancers (OR: 12.0) had the highest association with VTE (see Supplemental Table 5). Frequency of comorbidities and temporary provoking factors in patients with VTE and matched controls are shown in Supplemental Table 6.

### Multivariable Adjusted Odds Ratios of Different Cancer Types in Patients With VTE Compared to Matched Controls Without VTE

**Figure 4.**
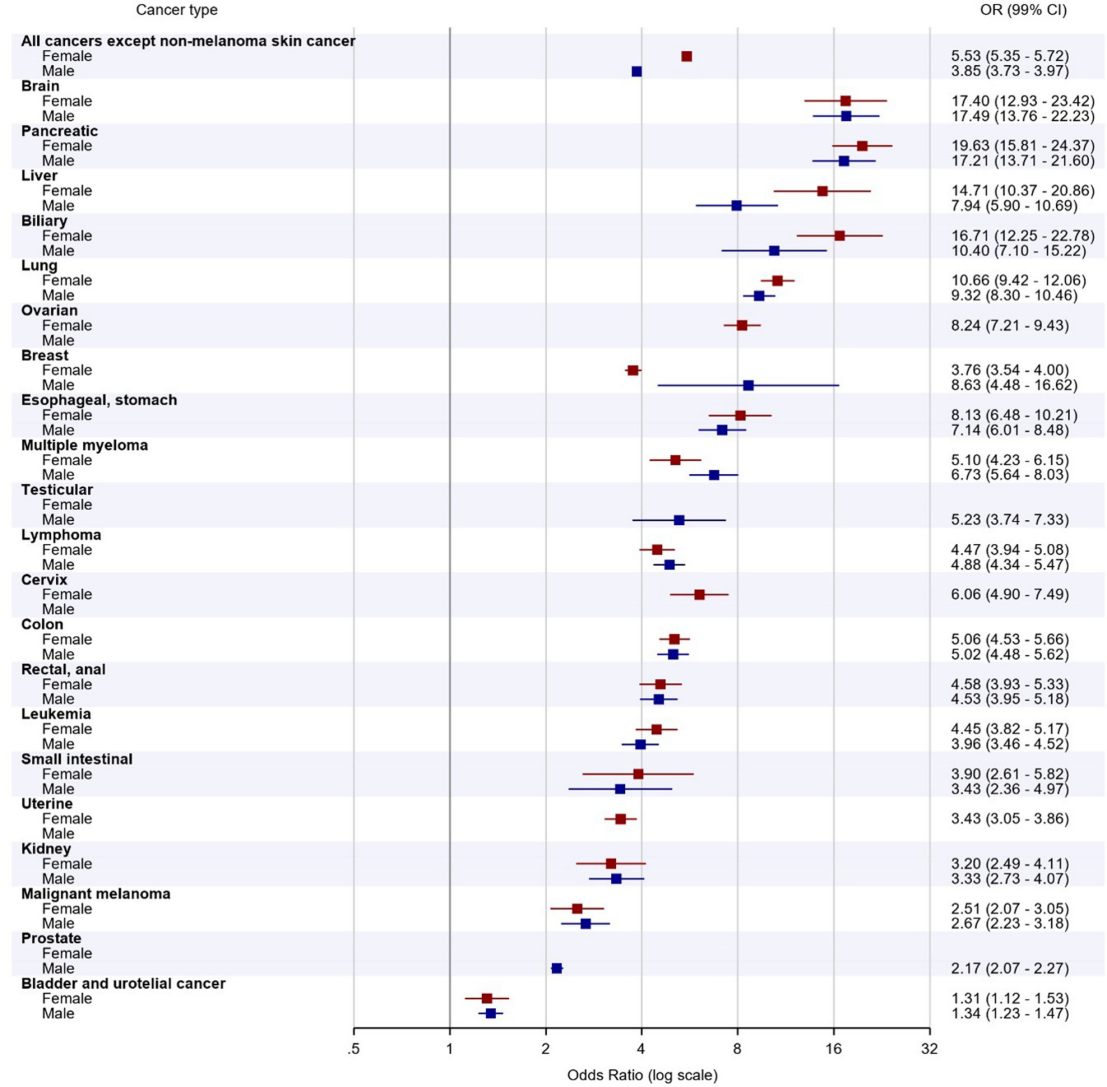
Multivariable aORs with 99% CIs for different cancer types in female and male patients with VTE. Abbreviations: aORs, adjusted odds ratios; VTE, venous thromboembolism.

After adjustment for comorbidities and temporary provoking factors, the aOR (99% CI) of cancer in patients with VTE was higher in women than in men (aOR 5.5 [99% CI 5.4-5.7] vs 3.9 [99% CI 3.8-4.0]) compared to their respective controls without VTE. After excluding sex-specific cancer forms, the aOR for women remained slightly higher than that for men (aOR 5.6 [99% CI 5.4-5.8] vs 5.0 [99% CI 4.8-5.1]). The only significant difference in aOR for VTE between men and women within separate cancer types was for breast cancer: aOR 8.6 (99% CI 4.5-16.6) for men versus 3.8 (99% CI 3.5-4.0) for women. Information on aOR for all cancers is found in Figure 4.

In females, the highest aORs were seen for pancreatic (aOR: 19.6 [99% CI: 15.8-24.4]), brain (aOR 17.4 [99% CI 12.9-23.4]), and biliary cancers (aOR 16.7 [99% CI 12.3-22.8]. In males, brain cancer had the highest (aOR 17.5 [99% CI 13.8-22.2]), followed by pancreatic (aOR 17.2 [99% CI 13.7-21.6]) and biliary cancers (aOR 10.4 [99% CI 7.1-15.2]) when comparing patients with VTE with matched controls without VTE.

In females, cancers with the lowest aORs included bladder/urothelial cancer (aOR 1.31 [99% CI 1.12-1.53]), malignant melanoma (aOR 2.51 [99% CI 2.07-3.05]), kidney cancer (aOR 3.20 [99% CI 2.49-4.11]), and uterine cancer (aOR 3.43 [99% CI 3.05-3.86]). Similar results were observed in males, with bladder/urothelial cancer having the lowest association with VTE (aOR 1.34 [99% CI 1.23-1.47]), followed by prostate cancer (aOR 2.17 [99% CI 2.07-2.27]), malignant melanoma (aOR 2.67 [99% CI 2.23-3.18]), and kidney cancer (aOR 3.33 [99% CI 2.73-4.07]).

## Discussion

In this large, registry-based, case-control study of patients with VTE and controls from the Swedish population, the most important finding was a fairly weak association between a diagnosis of VTE and a recent registration of kidney, bladder/urothelial, or uterine cancer. These findings are not in line with current guidelines^10,12^ or the Khorana risk score,^13^ which place kidney,^10,12^ bladder,^10,13^ and gynecological^10,13^ cancers in a high-risk category. However, these previous risk estimates were mainly based on studies in patients receiving chemotherapy.^10,13–16^ For kidney cancer, one study was based on only eight cases of cancer-associated VTE and showed a nonsignificant risk difference compared with controls.^17^

Two previous Danish studies support our findings to some extent.^1,9^ The most recent study found that uterine, bladder, and kidney cancers had an intermediate risk of VTE within 1 year of diagnosis, comparable to colon and rectal cancers.^1^ The earlier Danish study by Cronin-Fenton et al found that endometrial and kidney cancers were associated with a low risk of VTE, comparable to breast and prostate cancers, while urinary bladder cancer had an intermediate risk, comparable to colon cancer.^9^ Our findings for kidney and uterine cancers are also in line with data from a British study, in which bladder cancer was ranked among cancers with an intermediate risk.^11^

In several cancers, such as bladder/urothelial, kidney, and uterine cancers, thrombogenicity is dependent on the cancer stage. Data from The California Cancer Registry showed that many patients with bladder, kidney, or uterine cancers had localized disease at the time of diagnosis and a low risk of VTE, whereas metastasized bladder, uterine, or kidney cancers had the highest risk of VTE.^18^ These findings were recently confirmed in an updated analysis of the same registry.^19^ This is an important information for clinical practice: cancers associated with a high risk of VTE when they are regionally advanced or metastatic do not necessarily have a high risk of VTE at less advanced stages.

Cancers with a strong association to VTE in our study were in line with current guidelines^10^ and prior studies.^1,9,11,19^ Specifically, pancreatic, liver, biliary, brain, and ovarian cancers ranked among cancers with the highest association with VTE.

The differences in the risk increase in our study compared with those in previous studies are probably dependent of the study type.^1,8,9,11,19^ Most previous studies did not verify the VTE diagnosis with retrieval of anticoagulant treatment,^1,8,9,19^ which may have caused overestimation of the VTE incidence.^20^ The inclusion criteria also varied, with some studies including additional types of venous thrombosis such as splanchnic vein thrombosis^1,8,9^ and superficial vein thrombosis.^8,9^

The incidence of VTE with cancer, as presented in Figure 2, increased with age, peaking at approximately 85 years. Men had a higher incidence compared with women at older ages. The higher incidence for male VTE with cancer was expected because of their higher cancer incidence compared with women.^21^ Relative to the incidence of VTE in the total population, the higher incidence of VTE with a diagnosis of cancer in men compared with women was more pronounced. The higher incidence also extended to older ages.^7^

The higher risk of cancer in female patients with VTE than in male VTE cases was mainly caused by differences in risk of sex-specific cancers. The remaining difference after exclusion of these cancers was small and probably not of any clinical significance. There were no significant sex-related differences within different cancer types, except for breast cancer, where men were at higher risk compared to VTE-free controls than women. Male breast cancer is rare and is often diagnosed at an older age and later stage than female breast cancer, possibly influencing our results.^22^ Previous studies are in line with our data and found small, if any, sex differences in association between VTE and cancer. A large registry-based study of patients with cancer reported higher multivariable adjusted risk of hospital-associated VTE in women (4.3%) compared with men (4.0%), giving an OR of 1.14 (95% CI 1.12-1.16).^23^ No difference was seen in the large Danish registry-based studies after multivariable adjustment.^1,9^

## Strengths and Limitations

The major strength of this study is the very large study population, covering all patients with VTE and cancer in Sweden for more than 3 decades and matched controls from the general population. This provides sufficient power to conduct subanalyses on many different cancer types.

The largest limitation of the study is the lack of information on cancer stages. It is well known that the VTE risk is higher in more advanced disease.^1,18,19^ However, our data did not allow us to differentiate cancer stages or adjust for antitumoral therapy, only the presence or absence of a cancer diagnosis.

Other potential limitations of the study include the lack of external validation of the VTE diagnoses. The validity of VTE diagnoses in Swedish patient registries has been questioned, particularly for outpatient diagnoses.^20,24^ Therefore, the diagnoses after July 2005 were verified by retrieval of anticoagulant drugs, and the outpatient diagnoses before that date only included patients with first DVT or PE and subsequent same diagnosis within 3 months. Swedish VTE diagnoses before 1998 were previously shown to have good validity.^25^

The results of this study are likely to be generalizable to populations with similar thrombotic risk and accessibility to healthcare to the Swedish population. However, the generalizability to other groups is unknown.

In conclusion, in this large registry-based study, we describe age-specific and sex-specific incidence of VTE with recent cancer and ORs for various cancers in patients with VTE compared to matched controls without VTE. We found a strong association between VTE and pancreatic, and brain and biliary cancers. We found a weak association with bladder/urothelial, kidney, and uterine cancers for VTE, in contrast to current guidelines. This finding suggests that a proportion of patients in these cancer groups are at a fairly low risk of VTE, maybe related to the stage of cancer, but needs further research.

## Supplemental Material

sj-docx-1-cat-10.1177_10760296231158368 - Supplemental material for Prevalence of Cancer in Patients with Venous Thromboembolism: A Retrospective Nationwide Case-Control Study in Sweden Click here for additional data file. Supplemental material, sj-docx-1-cat-10.1177_10760296231158368 for Prevalence of Cancer in Patients with Venous Thromboembolism: A Retrospective Nationwide Case-Control Study in Sweden by Katarina Glise Sandblad, Per-Olof Hansson, Jacob Philipson, Ahmad Mahmoud, Per Karlsson, Annika Rosengren and Jan Sörbo in Clinical and Applied Thrombosis/Hemostasis
