# Supplementary material for: Prevalence of Cancer in Patients with Venous Thromboembolism: A Retrospective Nationwide Case-Control Study in Sweden
Source: Clin Appl Thromb Hemost. 2023 Feb 27;29:10760296231158368. doi: 10.1177/10760296231158368 (PMC9986905; doi:10.1177/10760296231158368)
Supplement: sj-docx-1-cat-10.1177_10760296231158368 - Supplemental material for Prevalence of Cancer in Patients with Venous Thromboembolism: A Retrospective Nationwide Case-Control Study in Sweden [file sj-docx-1-cat-10.1177_10760296231158368.docx]

**Supplementary appendix**

Prevalence of cancer in patients with venous thromboembolism: A retrospective nationwide case-control study in Sweden

Katarina Glise Sandblad^a,b^, P O Hansson^a, b^, Jacob Philipson^b^, Ahmad Mahmoud^a,b^, Per Karlsson^c^, Annika Rosengren^a,b^, Jan Sörbo^d^

^a^Department of Molecular and Clinical Medicine, Institute of Medicine, Sahlgrenska Academy, University of Gothenburg, Sahlgrenska University Hospital/Östra, Gothenburg SE 416 85, Sweden

^b^Department of Medicine, Geriatrics and Emergency Medicine, Region Västra Götaland, Sahlgrenska University Hospital/Östra, Gothenburg SE 416 85, Sweden

^c^Department of Oncology, Institute of Clinical Sciences, Sahlgrenska Academy, Sahlgrenska University hospital/ Sahlgrenska, Blå stråket 2, Gothenburg University, Gothenburg, SE 413 45, Sweden

^d^Department of Clinical Physiology, Region Västra Götaland, Sahlgrenska University Hospital/Östra, Gothenburg SE 416 85, Sweden

**Table of contents**

**Content Page**

Supplementary table 1: Information on included registers 3

Supplementary table 2: Diagnostic codes cancer. 4

Supplementary table 3: Diagnostic codes for multivariate adjustment. 5

Supplementary table 4: Age-and sex-specific incidence rates of CAT. 6

Supplementary table 5: Univariable odds ratios for various cancer types in

female and male VTE cases. 7

Supplementary table 6: Frequency of comorbidities and temporary

provoking factors in CAT-patients. 8

Supplementary statement 9

Supplementary Table 1. Information on included registers.

| **Registry** | **Content** |
| --- | --- |
| The Swedish Patient Register | Diagnostic and procedural codes of hospital-based care. Complete national coverage inpatient data since 1987. Hospital outpatient data since 2001. |
| The Swedish Cause of Death Register | Deaths of all persons registered in Sweden. Data since 1961. Including main and contributing causes of death and date of death. |
| The Total Population Register | Record of all Swedes since 1968. |
| The Prescribed Drug Register | All retrieved prescriptions since July 1, 2005. Data such as strength of medications, package size, date of retrieval |

Supplementary table 2.

Diagnostic codes for various cancer types.

| **Cancer type** | **ICD 8** | **ICD 9** | **ICD 10** |
| --- | --- | --- | --- |
| All cancers except non-melanoma skin cancer. | 140-209 NOT 173 | 140-208 NOT 173 | C00-C97 NOTC44 |
| Esophageal, stomach | 150,151 | 150, 151 | C15, C16 |
| Small intestinal | 152 | 152 | C17 |
| Colon | 153, 154,01 | 153, 154A | C18,C19 |
| Rectal, anal | 154 NOT 154,01 | 154 NOT 154A | C20,C21 |
| Pancreatic | 157 | 157 | C25 |
| Liver | 155 | 155 | C22 |
| Biliary | 156 | 156 | C23, C24 |
| Lung | 162 NOT 162,01 | 162 NOT 162 A | C34 |
| Brain | 191 | 191 | C71 |
| Malignant melanoma | 172 | 172 | C43 |
| Kidney | 189,00 | 189A | C64 |
| Bladder and urothelial cancer | 188, 189,11-189,99 | 188, 189B-W (NOT 189A) | C65-C67 |
| Uterine | 182 | 182 | C54 |
| Ovarian | 183 | 183 | C56 |
| Cervix | 180,99 | 180 | C53 |
| Leukemia | 204-207 | 204-208 | C91-95 |
| Lymphoma | 200-202 NOT 202,93 | 200-202 | C81-C88 |
| Multiple myeloma | 203 | 203 | C90 |
| Prostate | 185 | 185 | C61 |
| Testicular | 186 | 186 | C62 |
| Breast | 174 | 174 | C50 |

Supplementary table 3.

Diagnostic codes used for multivariable adjustment.

| **Diagnosis** | **ICD-9** | **ICD-10** |
| --- | --- | --- |
| **Comorbidities** | | |
| **Heart Failure** | 428 | I50 |
| **Ischemic heart disease** | 410-414 | I20-I25 |
| **Atrial fibrillation** | 427D | I48 |
| **Ischemic stroke** | 434, 436 | I63, I64 |
| **Hemorrhagic stroke** | 431-432 | I60-I62 |
| **COPD** | 490-492 | J40-J44 |
| **Inflammatory bowel disease** | 555, 556 | K50-K52 |
| **Temporary provoking factors within 3 months before VTE** | | |
| **Major surgery** | 0100-0699, 0800-0999, 2800-2898, 3000-3199, 3410-3599, 3800-3899, 4000-5699, 6000-7899, 8000-8799 , 8802-8851 | KVÅ A-B ,F-H, J-N, P |
| **Lower extremity fracture** | 820-829 | S72, S82, S92, T12 |
| **Trauma** | E 807-E849, E880-E887, E890-E899, E900-E909, E910-E939, E950-E969 | V01-X59 |

Supplementary table 4.

Age-and sex-specific incidence rates of VTE with cancer diagnosis at or before the date of VTE-diagnosis on a population level.

|  | **All** | | | **Men** | | | **Women** | | |
| --- | --- | --- | --- | --- | --- | --- | --- | --- | --- |
| **Age group** | **VTE index cases** | **Studied population years** | **per 100K** | **VTE index cases** | **Studied population years** | **per 100K** | **VTE index cases** | **Studied population years** | **per 100K** |
| 0-19 | 47 | 67 458 161 | 0.07 | 23 | 34 654 072 | 0.07 | 24 | 32 804 089 | 0.07 |
| 20-29 | 229 | 37 058 791 | 0.62 | 126 | 18 966 074 | 0.66 | 103 | 18 092 717 | 0.57 |
| 30-39 | 533 | 38 037 318 | 1.40 | 261 | 19 441 902 | 1.34 | 272 | 18 595 416 | 1.46 |
| 40-49 | 1 785 | 38 782 175 | 4.60 | 707 | 19 724 447 | 3.58 | 1 078 | 19 057 728 | 5.66 |
| 50-59 | 4 916 | 35 088 835 | 14.01 | 2 238 | 17 690 234 | 12.65 | 2 678 | 17 398 601 | 15.39 |
| 60-69 | 11 229 | 29 948 196 | 37.49 | 5 736 | 14 669 597 | 39.10 | 5 493 | 15 278 599 | 35.95 |
| 70-79 | 15 308 | 22 436 235 | 68.23 | 8 033 | 10 197 978 | 78.77 | 7 275 | 12 238 257 | 59.44 |
| 80-89 | 9 441 | 11 591 389 | 81.45 | 4 674 | 4 396 243 | 106.32 | 4 767 | 7 195 146 | 66.25 |
| 90- | 1 197 | 2 060 500 | 58.09 | 540 | 563 124 | 95.89 | 657 | 1 497 376 | 43.88 |

Supplementary table 5.

Univariable odds ratios for various cancer types in female and male VTE cases.

| **Cancer** | **Female** | **Male** |
| --- | --- | --- |
| **All cancers** | 7.41 | 5.25 |
| **Brain** | 28.93 | 31.32 |
| **Pancreatic** | 22.47 | 20.9 |
| **Biliary** | 19.47 | 14.01 |
| **Liver** | 16.49 | 10.63 |
| **Lung** | 13.71 | 11.97 |
| **Ovarian** | 11.57 |  |
| **Esophageal stomach** | 10.64 | 10.02 |
| **Cervix** | 9.16 |  |
| **Testicular** |  | 8.75 |
| **Colon** | 7.8 | 7.7 |
| **Rectal anal** | 6.65 | 6.86 |
| **Breast** | 5.27 | 8.27 |
| **Multiple myeloma** | 5.07 | 6.87 |
| **Uterine** | 5.35 |  |
| **Small intestinal** | 5.63 | 5.46 |
| **Lymphoma** | 5.3 | 5.55 |
| **Kidney** | 5.03 | 4.97 |
| **Leukemia** | 4.83 | 4.43 |
| **Malignant melanoma** | 2.82 | 2.99 |
| **Bladder and urotelial cancer** | 2.84 | 2.71 |
| **Prostate** |  | 2.89 |

Supplementary table 6.

Patients with VTE with cancer diagnosis at or before the date of VTE-diagnosis and their matched controls, frequency of comorbidities (diagnosed within a year before or on the same date as a VTE-diagnosis) and temporary provoking factors (diagnosed within a year before or on the same date as a VTE-diagnosis).

|  | **Study group** | | | | | |
| --- | --- | --- | --- | --- | --- | --- |
|  | **All** |  | **Women** |  | **Men** |  |
|  | **case** | **control** | **case** | **control** | **case** | **control** |
|  | **(n=44,685)** | **(n=177,716)** | **(n=22,347)** | **(n=88945)** | **(n=22338)** | **(n=88771)** |
|  | n, % | n, % | n, % | n, % | n, % | n, % |
| **Comorbidities (within 1 year)** |  |  |  |  |  |  |
| Heart failure | 3987 (8.9%) | 4276 (2.4%) | 1801 (8.1%) | 1641 (1.8%) | 2186 (9.8%) | 2635 (3.0%) |
| Ischemic heart disease | 4197 (9.4%) | 6066 (3.4%) | 1570 (7.0%) | 2021 (2.3%) | 2627 (11.8%) | 4045 (4.6%) |
| Atrial fibrillation/flutter | 3703 (8.3%) | 5089 (2.9%) | 1534 (6.9%) | 1849 (2.1%) | 2169 (9.7%) | 3240 (3.6%) |
| Ischemic stroke | 1640 (3.7%) | 2125 (1.2%) | 723 (3.2%) | 849 (1.0%) | 917 (4.1%) | 1276 (1.4%) |
| Haemorrhagic stroke | 339 (0.8%) | 273 (0.2%) | 147 (0.7%) | 97 (0.1%) | 192 (0.9%) | 176 (0.2%) |
| Chronic obstructive pulmonary disease | 2181 (4.9%) | 1991 (1.1%) | 953 (4.3%) | 849 (1.0%) | 1228 (5.5%) | 1142 (1.3%) |
| Inflammatory bowel disease | 651 (1.5%) | 715 (0.4%) | 329 (1.5%) | 356 (0.4%) | 322 (1.4%) | 359 (0.4%) |
| **Temporary provoking factors (within 3 months)** | | |  |  |  |  |
| Major surgery | 15831 (35.4%) | 5458 (3.1%) | 8158 (36.5%) | 2557 (2.9%) | 7673 (34.3%) | 2901 (3.3%) |
| Trauma | 670 (1.5%) | 587 (0.3%) | 369 (1.7%) | 314 (0.4%) | 301 (1.3%) | 273 (0.3%) |
| Lower extremity fracture | 846 (1.9%) | 592 (0.3%) | 487 (2.2%) | 369 (0.4%) | 359 (1.6%) | 223 (0.3%) |

Supplementary statement

Data from national health registries in Sweden can be obtained after ethical approval by contacting the National Board of Health and Welfare and requesting data needed for the research conducted.
